# Palatal plane inclination on vertical growth pattern among Indians

**DOI:** 10.6026/973206300171126

**Published:** 2021-12-31

**Authors:** Seerab Husain, Arvind Sivakumar, Sri Rengalakshmi

**Affiliations:** 1 Department of orthodontic and Dentofacial orthopaedics, Saveetha Dental College and Hospital, Saveetha Institute of Medical and technical Sciences, Saveetha University, Chennai, India

**Keywords:** Inclination angle, mandibular plane, palatal plane, vertical growth pattern

## Abstract

The skeletal discrepancies in the vertical dimensions can either lead to a long face or a short face. The palatal plane inclination is one such contributing factor. The study sample comprised of 15 lateral cephalograms collected between the time period of June 2019 - March 2020 with 5 cephalograms belonging to skeletal Class I, II and III respectively. The inclination angle and mandibular plane angle were measured using the FACAD software. The obtained results were tabulated and statistically analysed using Pearson's correlation test to determine the correlation between the two variables. There was a statistically significant negative correlation between the skeletal malocclusions with a p value of 0.011. Thus, palatal plane inclination is not a major contributing factor for vertical growth pattern and it is suggestive of a multifactorial influence.

## Background:

The hard and soft tissue both influence the length of then face thus contributing to facial harmony [1]. The two most commonly encountered vertical facial discrepancies are the vertical and horizontal growth pattern [2]. They are also termed as hyperdivergent or long face syndrome by Schendel and hypodivergent or short face syndrome as termed by Opdebeck [3,4]. Long face syndrome may arise due to the following reasons such as Increased oral/nasal airflow ratio, Muscle weakness, and Resting tongue posture, Heredity [5,6-check with author]. They may have detrimental effects on the psychological aspect of an individual. Physical appearance is also a factor that affects their social acceptance, self-esteem and psychological well-being. The term growth pattern is commonly employed because of the path of mandibular rotation, which guides the growth in that direction, causing lengthening/shortening of the lower anterior facial height [7]. Vertical growth pattern is more common in patients exhibiting an open bite, who have a divergent jaw base. On the contrary, horizontal growth pattern is more common in patients exhibiting deep bite, who have convergent and parallel jaw bases [8]. Mandibular plane angle has been commonly used by many investigators as prognostic criteria for measuring the vertical discrepancies. However, this has been contradicted by scieller and bjork et al., who suggest that the mandibular plane is not a sole indicator as a high angle case can also have a backwards or forward mandibular growth pattern [9]. Furthermore, it has been termed that horizontal/vertical growth patterns could arise due to a combination of several conditions such as the dental height, inclination of the maxilla, rotation of the core of the mandible, etc. Palatal plane inclination is measured from a perpendicular to the palatal plane (ANS-PNS) dropped from S-N plane. This is indicative of the inclination of maxilla, which could either be clockwise or anticlockwise. Therefore, it is of interest to investigate the correlation between palatal plane inclination and vertical growth pattern in Indian population.

## Materials & Methods:

The study was set up in the Saveetha institute of medical and technical sciences (SIMATS), chennai. Lateral cephalograms were collected from the Dental Information Archiving Software database spanning a time period of June 2019 - March 2020. Lateral cephalograms taken at a proper natural head position, with an FMA of more than 35°, without any missing teeth or any skeletal asymmetry were chosen for this study. Lateral cephalograms of poor quality and those of syndromic patients were not included in this study. 15 lateral cephalograms were randomly collected from the database, among which, 5 radiographs each were selected from Class I, II and III skeletal bases based on ANBo. Ethical clearance was obtained from the institutional review board. The lateral cephalograms were uploaded to the FACAD software for digital cephalometric tracing and analysis. The points marked on the radiographs were: Sella entry (Se), Soft tissue nasion (N'), Anterior nasal spine (ANS), Posterior nasal spine (PNS), Orbitale (Or), Porion (Po), Gonion (Go) and Menton (Mn). The angular values of Inclination angle and mandibular plane (MP) angle were measured. Inclination angle was measured from a perpendicular drawn from Se-N' line at N' to the palatal plane (ANS-PNS) and mandibular plane (MP) angle was measured between Frankfurt horizontal plane (Po-Or) and Mandibular plane (Go-Me). All the measured values from all the patients were tabulated and were subjected to statistical analysis using SPSS software version 23. Descriptive statistics showing mean and standard deviation and Pearson's correlation tests were performed to determine the correlation between inclination angle and mandibular plane angle in vertical growing individuals.

## Results and Discussion:

**Figure 1:**
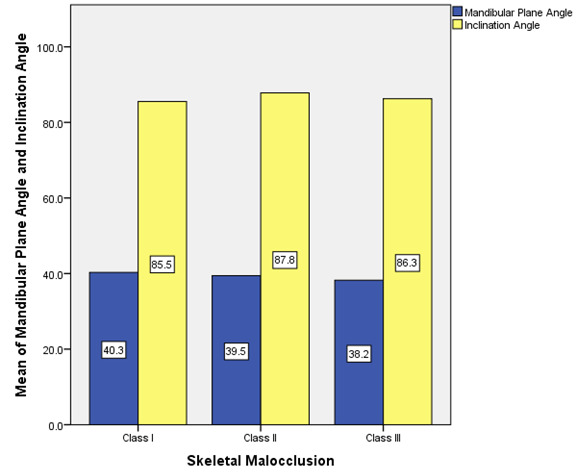
Bar chart showing mean value of mandibular plane angle and inclination angle in Class I, II and III malocclusion. Mean of the mandibular plane is 40.3° and the mean for inclination angle is 85.5° for Class I. For Class II, the mean of mandibular plane is 39.5° and the mean for inclination angle is 87.8°.The mean of mandibular plane is 38.2° and the mean for inclination angle is 86.3° for Class III. The mandibular plane angle is largest in Class I skeletal malocclusion, followed by Class II and then least in Class III. The inclination angle is maximum in Class II skeletal malocclusion, followed by Class III and then least in Class I. ( x axis - Skeletal relationship, y axis - Mean of mandibular plane angle and inclination angle; blue colour - mandibular plane angle, yellow colour - Inclination angle)

**Figure 2:**
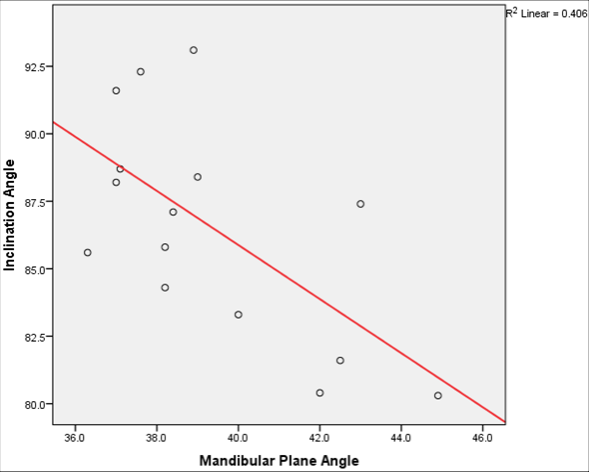
Scatter dot graph showing the results for Pearson's correlation test between mandibular plane angle and inclination angle in all 3 skeletal malocclusions. Figure shows a moderate negative correlation between the two angles, which is statistically significant as P value is 0.011. (x axis - mandibular plane angle; y axis - inclination angle)

Descriptive statistics showing mean of Mandibular Plane Angle and Inclination Angle in Class I, II and III malocclusions are depicted in (Figure 1). (Table 1 - see PDF) and (Figure 2) show results of Pearson's correlation test, which has a statistically significant negative correlation as P value is <0.05. Our team has been associated with various clinical trials [10], in vitro studies [11-16], Finite element studies [17-19] and a couple of prospective studies [20-24] spanning the last couple of years. This retrospective study was done with the data obtained from our vast university database. The need for further research in the field of orthodontics regarding the growth pattern of every individual stemmed the ideal for this study. Lateral cephalograms have been in use for a prolonged period of time for studies involving the growth and development of facial skeletal structures.[25] They are easy to interpret, economical and also ideal in catering to the needs of an orthodontic set up. Since the other alternatives like CBCT are too expensive and pose a threat of increased radiation exposure, lateral cephalograms were opted for this study. The aim of this research was to evaluate the correlation between inclination angle and mandibular plane angle in vertically growing patients.

Palatal plane inclination has been shown to have an impact on the growth pattern of many individuals. This is especially true in cases of open bite, where the posterior end of the palate is tipped down along with the maxillary molars, which acts as a fulcrum to rotate the mandible downwards and backwards [26]. The greater degree of angulation between the mandibular plane the palatal plane also necessitates the overgrowth of the dentoalveolar portion to mask the malocclusion [27]. This might not always be the case as in such situations, the malocclusion may manifest in the form of an open bite. A vertical pattern of growth also has detrimental effects on the musculature surrounding the chin, namely mentalis muscle. The backward mandibular rotation in a vertical growth pattern results in stretching of the mentalis muscle into forcefully closing the lower lip [28]. This inturn enforces its aberrant forces onto the lower teeth, and in turn, gets trapped underneath the upper anterior teeth, as in case of class II division I. Studies conducted by Cangialosi show palatal plane angle to be coincident with open bite and deep bite [29]. Although palatal plane is stable, it is highly variable in its inclination [30]. Larger basal plane angles are mostly common with open bite cases, which is due to both mandibular plane as well as palatal plane [31]. Studies show that the sagittal orientation of the palatal plane is stable, owing to the parallel natures of the growth curves, which is longitudinal in nature [32].

Other parameters like dental heights, gonial angle, occlusal inclination and mandibular plane angle are also commonly used as prognostic indicators for vertical discrepancies. Gonial angle, however, is said to decrease with age. This decrease was least in mandibular plane angle. From this study, it is evident that the palatal plane inclination has a moderate negative correlation with the mandibular plane. This might show a lesser influence of inclination angle on the vertical growth pattern. This finding is suggestive of the compensatory rotation of the mandible, to counter the effects of the palatal plane inclination. Also, considering the fact that for all the patients with a vertical growth pattern, palatal plane inclination might not be the only cause and this would in turn be suggestive of a multifactorial influence. There is still scope for precise evaluation in future studies, by increasing the sample size and also involving more parameters to measure [19]. Future studies should aim at involving all growth patterns with all the variables, which have an impact on the growth pattern of the jaw bases as vertical discrepancies are multi factorial.

## Conclusion:

Data shows that the inclination angle does not increase with mandibular plane angle. However, it has a significant negative correlation to mandibular plane angle. Thus, the inclination angle does not influence the vertical growth pattern in our population.

## References

[1] https://ijphrd.com/.

[2] Morris DH (1961). American Journal of Physical Anthropology..

[3] Schendel SA (1976). American Journal of orthodontics..

[4] opdebeeck H, Bell WH (1978). American Journal of orthodontics..

[7] Nahoum HI (1971). American Journal of orthodontics..

[8] Muller G (1963). Journal of Dental Research..

[9] Skieller V (1984). American Journal of orthodontics..

[10] Kumar VA (2014). International Journal of Dental Sciences and Research.

[11] Ravikumar D (2019). Journal of oral Biology and Craniofacial Research.

[12] Samantha C (2017). J Clin Diagn Res..

[13] Vikram NR (2017). J Clin Diagn Res..

[14] Kamisetty SK (2015). J Clin Diagn Res..

[15] Viswanath A (2015). Niger J Clin Pract..

[16] Felicita AS (2017). Dental Press J orthod..

[17] Rubika J (2015). World Journal of Dentistry..

[18] Sivamurthy G (2016). Progress in orthodontics..

[19] Jain RK (2014). Journal of Clinical And Diagnostic Research..

[20] Krishnan S (2018). Indian Journal of Dental Research..

[21] Ramesh Kumar KR (2011). Am J orthod Dentofacial orthop..

[22] Felicita AS (2017). Saudi Dent J..

[23] Felicita A (2012). Indian Journal of Dental Research..

[24] Dinesh SPS (2013). J Clin Diagn Res.

[25] Felicita AS, Sumathi Felicita A (2018). The Saudi Dental Journal..

[26] Isaacson JR (1971). Angle orthod..

[27] Sassouni V, Nanda S (1964). American Journal of orthodontics..

[28] Scott JH (1958). Am J orthod..

[29] Schudy FF (1965). Angle orthod..

[30] Cangialosi TJ (1984). American Journal of orthodontics..

[31] Sivakumar A (2021). Biology..

[32] Enlow DH, Bostwick J (1977). Plastic and Reconstructive Surgery..

